# Effects of conservation tillage strategies on soil physicochemical indicators and N_2_O emission under spring wheat monocropping system conditions

**DOI:** 10.1038/s41598-022-11391-6

**Published:** 2022-04-29

**Authors:** Jianyu Yuan, Lijuan Yan, Guang Li, Mahran Sadiq, Nasir Rahim, Jiangqi Wu, Weiwei Ma, Guorong Xu, Mengyin Du

**Affiliations:** 1 College of Forestry, Gansu Agricultural University, Lanzhou, 730070 China; 2 College of Agriculture, Gansu Agricultural University, Lanzhou, 730070 China; 3 Department of Soil and Environmental Sciences, University of Poonch Rawalakot, Rawalakot, 12350 AJK Pakistan

**Keywords:** Ecology, Climate sciences, Ecology, Environmental sciences

## Abstract

As one of the important greenhouse gas, nitrous oxide (N_2_O) has attracted much attention globally under climate change context. Agricultural practices are the main sources of greenhouse gas emissions. Nevertheless, scarcity of literature is available on the effects of different tillage measures on soil N_2_O emission under spring wheat (*Triticum aestivum L.*) ecosystem in the semi-arid area of the Loess Plateau. The main objective of the experimental study was to explore the influence of conservation tillage techniques on soil physicochemical properties, nitrous oxide emission and yield in the Northern semi-arid Dingxi region of China. Four treatments viz*.,* conventional tillage (CT), no tillage (NT), straw mulch with conventional tillage (TS) and stubble-return with no-till (NTS) were evaluated under randomized complete block design with three replications. Our results depicted that compared with conventional tillage, bulk density and water content of topsoil was increased and soil pH value was reduced under conservation tillage techniques. Conservation tillage NT, TS and NTS increased organic carbon, TN, MBN and NH_4_^+^-N and reduced the accumulation of NO_3_^–^N. Additionally, although the N_2_O emission under NT, TS and NTS was 8.95, 41.90 and 21.05% respectively higher than under T treatment, the corresponding wheat yield was 15.40, 31.97 and 63.21% higher than T treatment. Moreover, correlation analysis showed that soil moisture and temperature were the most significant factors affecting soil N_2_O emission. The NTS treatment pointedly increased crop yield without significantly increasing soil N_2_O emission. Consequently, based on economic and environmental benefits and considering N_2_O emission and crop yield, we suggest that NTS technique is the best conservation tillage strategy in the semi-arid environmental zone of the Loess Plateau of Dingxi China.

## Introduction

A series of ecological and environmental problems caused by global warming have become one of the important challenges facing mankind^1^. Due to the unreasonable use of natural resources by humans, the cycle of carbon and nitrogen in the biosphere has been accelerated^2^, the stability of carbon and nitrogen in the soil has been changed, and the concentration of greenhouse gases has continued to increase, resulting in the greenhouse effect, which has become a relevant focus of general attention in the field^3^. N_2_O is a major greenhouse gas, 80% to 90% of N_2_O in the atmosphere emits from cultivated soils annually, and cultivated soils are a significant source of emissions^4^. Studies have shown that the main pathways for the production of N_2_O in agricultural soil are nitrification and denitrification processes. Among them, environmental factors (temperature, moisture, pH, etc.) and soil carbon and nitrogen components are the key factors affecting N_2_O emissions^5^. Carbon and nitrogen components in the soil are transformed, absorbed and released by mineralization, nitrification and denitrification in the soil, which directly affect the global carbon and nitrogen balance, consequently affecting the global climate^6^. However, there is still a lack of systematic research on the analysis of soil N_2_O emission process and the relationship between environmental factors and N_2_O emission under different tillage methods. Consequently, exploring the effect of tillage measures on the regulation of soil N_2_O emissions and the effect of various regulatory mechanisms and environmental factors on N_2_O emissions are of great significance for promoting nitrogen balance, mitigating greenhouse gas emissions, and inhibiting climate warming. This could also provide a theoretical basis for the sustainable development of agroecosystems.

The Loess Plateau is located in the northern part of central China. Due to its increasingly severe ecological environment and complex geographical conditions, it has become one of the key research areas in ecology and related scientific hotspots^7^. In recent years, due to continuous human interference and unhealthy farming methods, the environmental problems such as soil erosion, poor soil fertility and increased greenhouse gas emissions in the Loess Plateau have become increasingly prominent. At present, the research on N_2_O emission from agricultural soils mainly focuses on fertilization, irrigation and intercropping. Scheer et al.^8^ found that irrigation, nitrogen application and soil surface temperature had a great impact on N_2_O emission from cotton soil in arid areas. The increase of irrigation and nitrogen fertilizer application could significantly increase N_2_O emission. Zhang et al.^9^ found that in a wheat maize rotation system, comparing shallow tillage and deep tillage, the global warming potential was greatest under the organic fertilizer treatment, and the organic fertilizer addition treatment increased the emission of N_2_O and reduced the absorption of CH_4_. Liu et al.^10^ found that the addition of nitrogen and water significantly increased the emission of N_2_O. The interaction of soil water and nitrogen had a significant impact on N_2_O emission. Besides soil water and nitrogen, temperature also significantly affected soil N_2_O emission. Hu et al.^11^ found that straw retention had no significant effect on N_2_O emission, but N_2_O emission increased by 72.5 ~ 311.1% due to the increase of AOA-amoA, AOB-amoA, nirK and nirS abundance. Furthermore, straw returning remarkably increased greenhouse gas intensity (GHGI), while nitrogen fertilization significantly reduced GHGI. These studies reveal the relationship between management practices and N_2_O emission to a certain extent, but in human activities, cultivation methods are the most important factor affecting soil nutrient status and greenhouse gas emission, especially conservation tillage^12^. Conservation tillage such as minimum tillage, no tillage and straw mulching reduces soil erosion and soil disturbance, retains soil nutrients, and then reduces greenhouse gas emissions, thus achieving nitrogen accumulation and emission reduction^13^. Consequently, it is necessary to explore the influence of conservation tillage on soil physicochemical quality indicators and N_2_O emission on the semi-arid Loess Plateau zone, which plays a vital role in crop productivity and ecological sustainability in the region.

In order to achieve the objective of this study, we took the spring wheat field in the Loess Plateau as the research object, and used traditional tillage method as a control. Three types of conservation tillage were tested to study the effects of different tillage measures on the soil nitrogen composition and N_2_O emission flux on the Loess Plateau. This study will provide theoretical basis and basic data on the dynamics of soil nitrogen and N_2_O emissions on the Plateau. The specific objectives of this study were: (1) To assess the changes of soil physical and chemical properties under different tillage measures (2) To explore the effect of conservation tillage on N_2_O emission flux and spring wheat yield (3) To examine the correlation between soil physicochemical attributes and N_2_O emission. We hypothesized that conservation tillage techniques would manifest improve soil physical, chemical quality attributes and wheat productivity as well as reduce N_2_O emission.

## Materials and methods

### Site description

**Figure 1:**
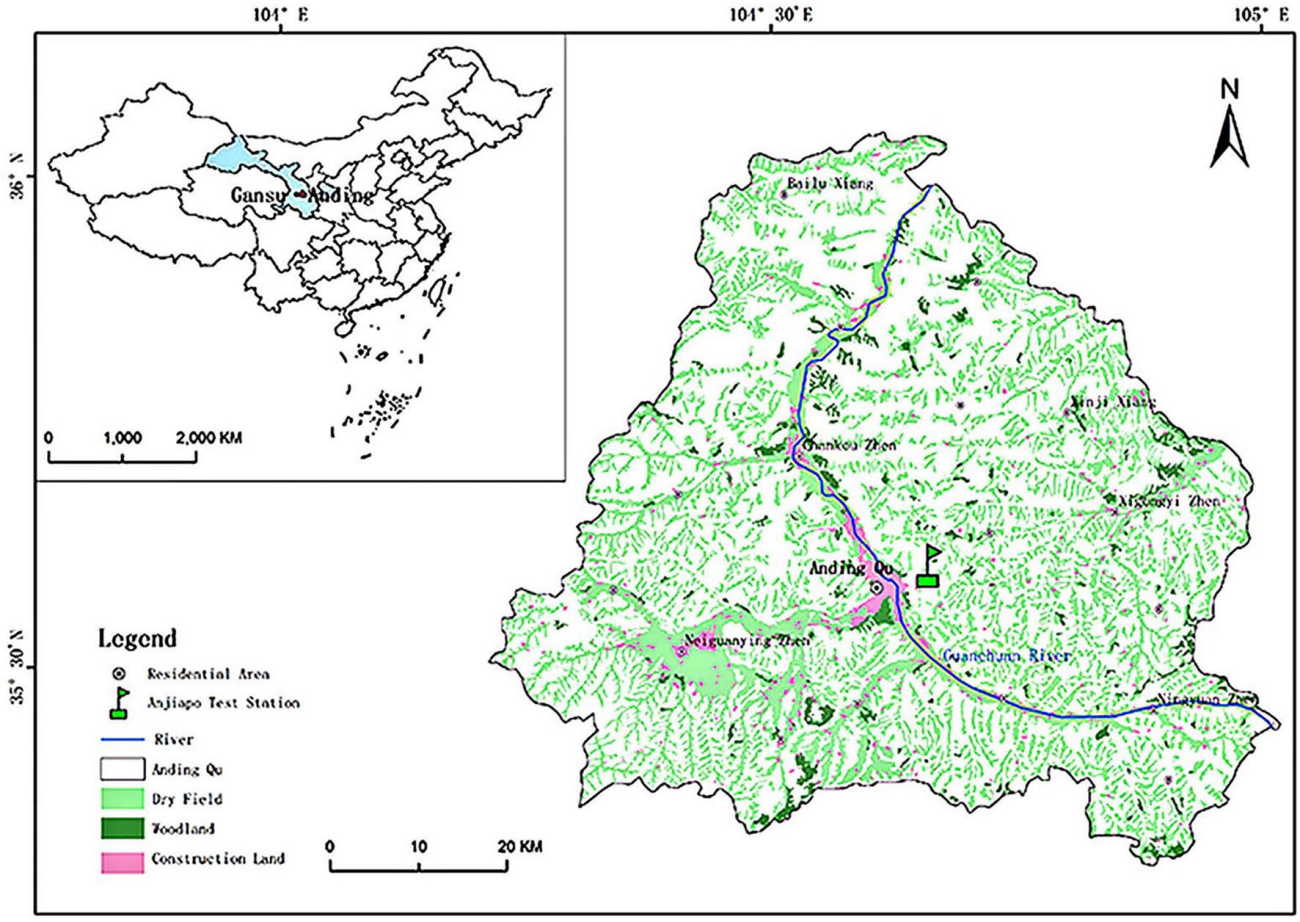
The geographical location map of the study area. Apply ArcGIS 10.2 Software production. The basic geographic information data comes from the resource and environmental science and data center (http://www.resdc.cn/).

The experimental study area is located in the dry agriculture comprehensive experimental station of Gansu Agricultural University, in Anjiapo village, Anding District, Dingxi City, Gansu Province (Fig. 1). The site is a typical hilly plateau, with a Loessal soil type which has a deep soil layer and gullies with an average elevation of 2000 m^14^. It is a temperate semi-arid zone with an annual average temperature of 6.4 °C^15^. The average ≥ 0 °C accumulated temperature is 2933.5 °C·d^16^, and the annual average ≥ 1 °C accumulated temperature is 2239.1 °C·d. The average annual precipitation is 385 mm. The average precipitation during the growth period of spring wheat is 189.86 mm, the maximum precipitation during the growth period is 286.70 mm, and the minimum precipitation during the growth period is 56.10 mm. The average annual solar radiation is 141.60 kJ cm^−2^, the average annual evaporation is 1540.00 mm^17^, and the site is a typical semi-arid rain fed agricultural area^14^. The major soil physicochemical quality indicators taken from the different soil layers in March 2020, before this research, are presented in Table 1. Averaged across three soil layers (0–10 cm, 10–20 cm, and 20–40 cm), the pre-planting soil NO_3_^−^-N, NH_4_^+^ -N, TN, TP, TK, SOC, C:N ratio, pH, SWC, electrical conductivity, bulk density, porosity, water storage, and temperature were 25.82 mg kg^−1^, 10.08 mg kg^−1^, 0.59 g kg^−1^, 0.41 mg kg^−1^, 18.46 g kg^−1^, 5.79 g kg^−1^, 9.82, 8.40, 14.95%, 0.35 dSm^−1^, 1.41 g cm^−3^, 46.79%, 48.83 mm, and 6.22 °C, respectively, and the texture of soil was sandy loam.

**Table 1:** Soil physical and chemical properties before sowing.

Soil property	Soil layer (cm)			Measurement method
	0–10	10–20	20–40	
NO_3_^–^N (mg kg^−1^)	25.72	25.77	25.88	Colorimetric method
NH_4_^+^-N (mg kg^−1^)	10.15	10.08	10.02	Colorimetric method
TN (g kg^−1^)	0.62	0.59	0.56	Semimicro–Kjeldahl method
TP (mg kg^−1^)	0.44	0.41	0.38	Colorimetric method
TK (g kg^−1^)	18.47	18.52	18.40	Colorimetric method
SOC (g kg^−1^)	5.93	5.81	5.64	Walkley–Black dichromate oxidation
C:N ratio	9.56	9.84	10.07	SOC/TN
pH	8.37	8.40	8.44	pH meter
ECe (dSm^−1^)	0.35	0.38	0.32	EC meter
B.D (g cm^−3^)	1.38	1.41	1.44	Core sampler method
P (%)	47.92	46.79	45.66	(1 − (BD/P)) × 100 equation
SWC (%)	15.65	14.76	14.46	Oven-dry method
SWS (mm)	21.59	41.62	83.28	SWC × BD × d/ρw
ST (°C)	6.40	6.22	6.04	Geothermometer
Soil texture	Sandy-loam			Hydrometer method

P.D: particle density = (2.65 g cm^−3^).

*ρw* density of water, *d* soil depth, *NO*_*3*_^*–*^*N* nitrate nitrogen, *NH*_*4*_^*+*^*-N* ammonium nitrogen, *TN* total nitrogen, *TP* total phosphorous, *TK* total potassium, *SOC* organic carbon, *C:N ratio* soil carbon and nitrogen ratio, *pH* soil pH, *ECe* electrical conductivity, *B.D* soil bulk density, *P* soil porosity, *SWC* gravimetric soil water content, *SWS* soil water storage, *ST* soil temperature.

Before the study, the research field was bare. The Loess Plateau of China, particularly in the Dingxi region, has a long history of spring wheat farming. In meteorological view point, on the basis of long-term (50-years) weather data analysis; in 2020 the annual temperature was 7.12 °C and total annual rainfall was 512 mm which was adequate for wheat growth and development. The total monthly rainfall and average monthly temperature are shown in Table 2.

**Table 2:** Climatic conditions of the study site during 2020.

Total annual rainfall = 512.50 mm	Average annual temperature = 7.23 °C	
Months	Rainfall (mm)	Temperature (°C)
Janvery	7.5	− 4.66
February	4.7	− 1.62
March	14.3	3.86
April	13.1	7.92
May	84.9	13.64
June	72.4	17.10
July	91.9	18.28
August	138.2	17.12
September	45.2	12.93
October	26.2	6.91
November	10.5	1.52
December	3.6	− 6.40

Climatic data contains total monthly rainfall and average monthly temperature.

### Experimental design

This study was conducted as part of continuing research initially set up in 2016 with different tillage measures. The results of the study in 2020 are shown in this paper. Four tillage treatments were established in a randomized complete block design. The treatments included traditional tillage (T), no-tillage (NT), traditional tillage with straw mulching (TS) and no-tillage with straw mulching (NTS). There were 3 repetitions for each treatment, and a total of 12 sample plots, the sample plot area is 4 m × 6 m. The description of treatments is shown in (Table 3). The spring wheat "Dingxi 42" cultivated locally was selected as the test variety (cv: Dingxi 42, the approval number: ganshengmai 2014004)^18^. Sowing was conducted in April 4 and harvested in August 5, 2020. In the tilled plots, soils were tilled at two different times by manual inversion with shovels to a depth of 20 cm; first in October of the previous year and again in March just before planting. Glyphosate (30%) herbicide was applied to control weeds in the plots. Wheat straw (dry weight of 3.75 ton ha^−1^) was spread uniformly on all straw-treated plots immediately after planting. The sowing rate of each plot was 187.5 kg·hm^−2^, and the row spacing was 25 cm. We used 150 kg·hm^−2^ Di-ammonium phosphate (N + P_2_O_5_) and 62.5 kg hm^−2^ urea as the base fertilizer^16^. In order to avoid the marginal effect between the plots, they were separated by a 0.5 m-wide isolation belt. All other field management and protection measures were consistent with local cultivation practices. A 50 cm × 50 cm × 20 cm stainless steel base was buried in each plot, with a sealed water tank on the top, which was used for the determination of soil N_2_O^19^. The selection of farmland and all studies on the test site comply with relevant institutional, national and international norms and legislation.

**Table 3:** Description of treatments of the experiment.

Treatment	Operation
T	The field was ploughed 3 times and harrowed twice after harvesting
NT	No-tillage without straw mulching throughout the experiment. Sowing and fertilization were completed by no-tillage planter by one time
TS	Tillage practice was as that of treatment T, but with straw incorporated at the first plough. All the straw from the previous crop was returned to the original plot immediately after harvesting and then incorporated into ground
NTS	Tillage practice was as that of treatment NT. The ground was covered with straw of previous crop from August to next March. All the straw from previous crop was returned to the original plot immediately after harvesting

### Sample collection and measurements

#### Determination of soil physical and chemical characteristics

Soil sampling was done concurrently with gas sampling periods during the spring wheat crop growth period. Five points were randomly selected in each test plot in the shape of "S", aboveground plants and surface litter were removed. Soil core sampler with a diameter of 5 cm was used to collect soil samples at 0–10 cm soil layer. Debris were removed and stored in a ziplock bag and placed in a sample box with an ice bag for cooling during transportation^20^. These samples were taken back to the laboratory for the determination of soil indicators. The oven-drying method was used for gravimetric soil water content determination. The cylindrical ring cutter was weighed first, and the fresh soil sample was then put in the ring, which was weighed and transferred to an oven. After drying to a constant weight at 105–110 °C, the weight of the dry soil was determined, and the percentage of water by weight was then calculated to obtain the soil water content^21^. The soil pH was measured by Potentiometric method (soil water ratio = 1:2.5)^22^, retrieved soil sample was naturally air dried without light. 10 g of air-dried soil sample was sieved by passing through 1 mm sieve, and put into a 100 ml beaker. Then 25 ml of distilled water was then added and mixed thoroughly and allowed to settle for 30 min. The pH value of the suspension was measured with a calibrated pH meter. The core sampler method was used for determination of soil bulk density^23^. Briefly, we used the cutting ring to retrieve the original soil, dried the soil at 105 °C, measured the dry weight of the soil, and then calculated the soil bulk density. The temperature of soil (0–10 cm) was measured by EM50 data logger.

#### Determination of soil organic carbon

Soil organic carbon (SOC) was determined by the Walkley–Black dichromate oxidation method^14^, using a mixture of potassium dichromate (K_2_Cr_2_O_7_) and sulfuric acid (H_2_SO_4_) to oxidize the organic matter, after which it was titrated against ferrous sulfate (FeSO_4_). The air-dried soil sample (0.1 g) was extracted with 7.5 ml of K_2_Cr_2_O_7_ and 7.5 ml of concentrated H_2_SO_4_ at 180 °C for 30 min.

#### Determination of soil nitrogen components

The content of TN in soil was determined by oxidation external heating titration and semi micro Kjeldahl method^24^. 1 g of air-dried soil sample was weighed. 8 ml of concentrated sulfuric acid was added to the sample and placed on the digestion furnace which was heated to milky white at 400 ℃. The whole solution was transferred to a 100 ml volumetric flask at constant volume, absorb 5 ml of supernatant with a pipette after the solution is cooled and clarified, put it into the digestion tube, add 4 ml of sodium hydroxide (10 mol/l) and 5 ml of boric acid solution for nitrogen determination, and titrate with dilute sulfuric acid solution.

The contents of NH_4_^+^-N and NO_3_^–^N in soil was determined by 2 mol/l KCL solution with water soil ratio of 5:1. After extraction, they are determined by flow analyzer^25^, weigh 10 g of a fresh soil sample that has passed through a 2 mm sieve into a 250 ml Erlenmeyer flask, add 50 ml of 2 mol/L KCL solution, shake on a reciprocating shaker for 1 h, filter the shaken soil suspension with a quantitative filter paper with a diameter of 12.5 cm Turbid liquid, the filtered soil extract uses a continuous flow analyzer to measure the content of soil NO3^–^N and NH_4_^+^-N.

The content of MBN in soil was determined on fresh soil samples (sieving < 2 mm) using the chloroform fumigation-extraction method^26^. The fumigated soil and non-fumigated soil (5 g, accurate to 0.001 g) were extracted with 20 ml 0.5 M of K_2_SO_4_ for 30 min on a shaker (180 rpm), absorb 10 ml of the leaching filtrate into the digestion tube, add 1.08 g of K_2_SO_4_-CuSO_4_-Se mixed catalyst (at the same time 0.2 g of nitrogen alloy can also be added to reduce nitrate nitrogen), add 4 ml of concentrated sulfuric acid; set 2 at the same time ~ 3 blanks; it is best to leave it overnight, the next day, digest it at low temperature (about 150 °C to remove water) in the digestion furnace, and then digest at high temperature (320 °C) until it is clarified, and then place it for 2 ~ 3 h. Then, the nitrogen content was measured by the semi-micro distillation method.

#### Determination of N_2_O in soil

Greenhouse gases were collected by static box gas collection method^27^, and greenhouse gases are collected every 20 days during the growth period of spring wheat. The gas flux measurements were conducted in quadruplicate and the mean value was calculated and analyzed. During sampling, an open bottom stainless-steel chamber (50 cm × 50 cm × 50 cm, equipped with two fans at the top powered by 12 v batteries to mix the air inside the chamber) with a rubber seal strip pasted on the open bottom part and placed over the collar to ensure tightness. Air samples (five in total) were drawn from inside the chamber right after chamber closure (T0) and every 10 min thereafter over a 40 min period using 100 ml gas-tight polypropylene syringes equipped with three-way stopcocks. The drawn sample was then injected into polyethylene coated aluminum bags via a rubber tube connected to the valve. Gas sampling usually occurred between 9 am and 12 pm. Fluxes measured within this period were found to be representative of the daily average flux on the test area^28^. Gas samples were immediately taken to the laboratory and analyzed within 3 days after sampling.

The gas samples were measured and analyzed by Agilent hp5890 gas chromatograph. The target gas mixture ratio of each group of 4 samples is linearly fitted to the corresponding sampling time interval. When the regression coefficient R^2^ > 0.75, it is regarded as valid data and the used to calculate the emission flux of the target gas^19^.

The calculation formula of N_2_O emission flux (F) is:

$$F = \rho \cdot h \cdot \frac{273}{{(273 + T)}} \cdot \frac{dc}{{dt}}$$

In the formula: F is the emission flux of gas, mg / (m^2^·h), A negative value indicates that the soil absorbs the gas, and a positive value indicates that the soil emits the gas. ρ is the gas density under standard conditions (g/cm^3^); h is the height of the sampling box (1.0 m); T is the temperature in the box at the time of sampling (℃); dc/dt is the rate of change of the gas concentration in the box.

The calculation formula of N_2_O cumulative emissions is:

$$M = \sum {\frac{{(F_{i + 1} + F_{i} ) \times (t_{i + 1} - t_{i} ) \times 24}}{2 \times 100}}$$

In the formula: M is the cumulative emission of gas in the whole growth period (kg hm^−2^); F is the gas emission flux (mg m^−2^ h^−1^); i is the number of samplings; t is the sampling time (day).

#### Determination of yield of spring wheat

The yield of spring wheat was measured at harvest. During the harvest period, three rows of wheat with uniform growth were harvested in each plot and manually threshed. The wheat grains in each plot are put into bags and brought back to the laboratory. The grain yields were determined by oven-drying at 105 °C for 45 min^29^. Finally, we estimated the yield of the plot through extrapolation from wheat yield per 3 rows to yield per hectare.

### Statistical analysis

We used SPSS 20.0 software for statistical analysis of the data, and used Excel and origin to draw the graphs. Significant differences of variables under different tillage measures were analyzed by one-way analysis of variance (ANOVA) and least significant difference (LSD) at *P* ≤ 0.05. Pearson correlation analysis was used to describe the correlation between various factors.

## Results

### Soil physical quality indicators under conservation tillage strategy

The bulk density and gravimetric soil water content of spring wheat (0–10 cm) soil under different tillage measures were analyzed (Table 4). The results showed that the three conservation tillage treatments increased soil bulk density and soil water content. Soil bulk density was the largest under NTS treatment and the smallest under T treatment, and there were significant differences among the treatments (*P* < 0.05), and the soil water content was the largest under the TS treatment and the smallest under the T treatment.

**Table 4:** Changes of soil physical properties of 0-10 cm spring wheat under different treatments.

Treatment	Bulk density (g cm^−3^)	Gravimetric soil water content (%)
T	1.15 ± 0.072 D	8.62 ± 0.04 C
NT	1.18 ± 0.076 B	8.71 ± 0.09 BC
TS	1.16 ± 0.073 C	9.41 ± 0.09 A
NTS	1.20 ± 0.029 A	8.98 ± 0.10 B

Different capital letters indicate significant differences among different treatments (*P* < 0.05).

**Figure 2:**
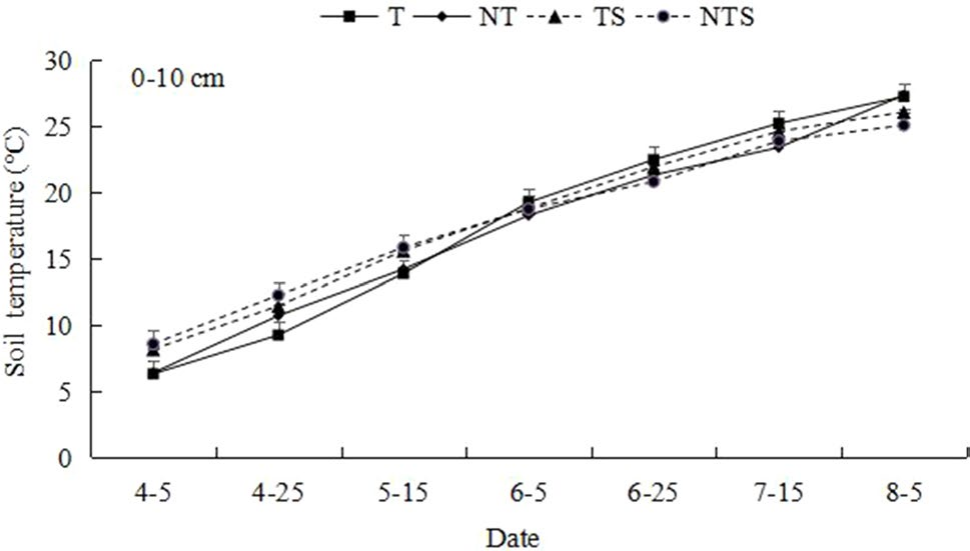
Temperature of 0–10 cm soil layer under different tillage measures.

It can be seen from Fig. 2 that the soil temperature of 0–10 cm under different cultivation measures had obvious seasonal changes. After the spring wheat, soil temperature continued to rise until the final harvest. From the perspective of different treatments, in the early stage of spring wheat growth, the soil temperature under conservation tillage (NT, TS, NTS) was higher than that under traditional tillage (T), with NTS treatment being the highest. But from June 25th until maturity, the temperature was the highest under the traditional tillage (T) treatment and the lowest under the no-tillage with mulch (NTS) treatment.

### Response of soil chemical quality indicators to conservation tillage technique

The soil chemical quality indexes under different tillage measures were analyzed (Table 5). The results showed that compared with traditional tillage, the three conservation tillage treatments increased soil organic carbon and reduced soil pH. The soil organic carbon of NTS treatment was the largest and T treatment was the smallest. There was significant difference among the treatments (*P* < 0.05). Soil pH value showed that NTS treatment was significantly lower than other treatments (*P* < 0.05).

**Table 5:** Changes of soil chemical properties of 0–10 cm spring wheat under different treatments.

Treatment	pH value	Organic carbon (g/kg)
T	8.32 ± 0.017 B	7.47 ± 0.09 C
NT	8.34 ± 0.032 A	7.94 ± 0.09 B
TS	8.29 ± 0.032 C	8.17 ± 0.04 B
NTS	8.26 ± 0.034 D	9.14 ± 0.05 A

Different capital letters indicate significant differences among different treatments (*P* < 0.05).

**Figure 3:**
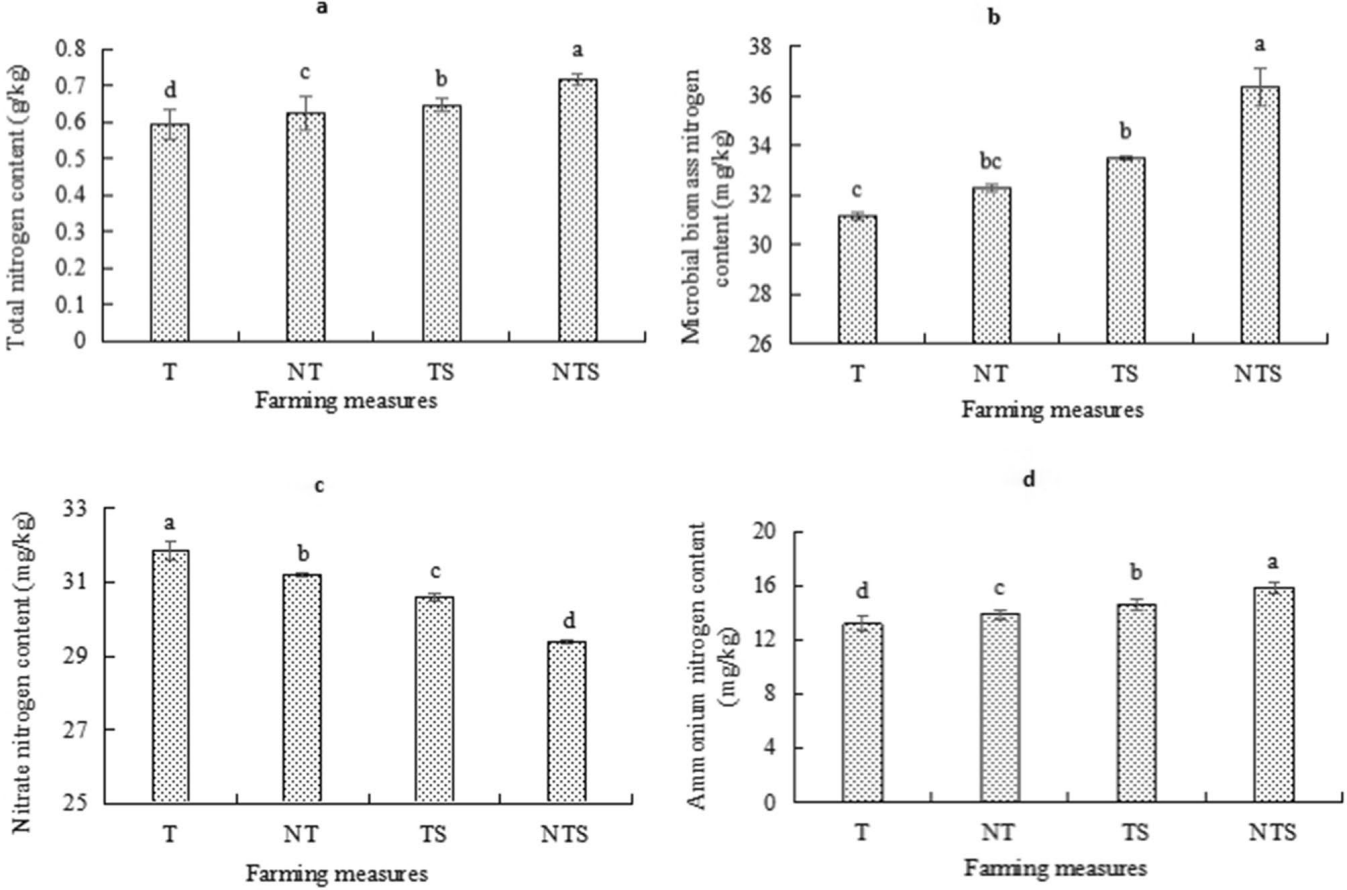
Nitrogen content in 0–10 cm soil of spring wheat under different tillage measures. Different lowercase letters indicate significant differences among different treatments (*P* < 0.05).

The contents of TN, NO_3_^–^N, NH_4_^+^-N and MBN in 0-10 cm soil during the whole growth period of spring wheat under different tillage measures are shown in Fig. 3. There were significant differences in soil TN content among treatments (*P* < 0.05). The soil TN content under conservation tillage treatment was significantly higher than that under traditional tillage, with NTS treatment being the highest (0.72 g kg^−1^), which was 21.1%, 15.3% and 11% higher than T, NT and TS treatments respectively. It shows that both no-tillage and straw mulching treatments can increase soil nitrogen content, and the combination of the two has a better effect. Soil MBN followed a similar trend as TN, with the maximum under NTS treatment (36.37 mg kg^−1^) and the minimum under T treatment (31.12 mg kg^−1^), and there were significant differences among treatments. NTS, TS and NT treatments were 16.87%, 7.54% and 3.75% higher than T treatment respectively.

In general, the content of NO_3_^-^N in the soil of the study area was much greater than that of NH_4_^+^-N (Fig. 3). The content of NO_3_^–^N in soil was the largest under T treatment (31.85 mg kg^−1^), and the smallest under NTS treatment (29.4 mg kg^−1^). The specific performance was T > NT > TS > NTS, and there were significant differences between the treatments (*P* < 0.05). On the contrary, the content of soil NH_4_^+^-N was the largest under NTS treatment (15.85 mg kg^−1^) and the smallest under T treatment (13.17 mg kg^−1^). The no-tillage, traditional tillage with straw mulching and no-tillage with straw mulching were 5.2%, 11% and 20.3% higher than traditional tillage, indicating that conservation tillage was conducive to the accumulation of soil NH_4_^+^-N content.

### Impacts of conservation tillage on N_2_O emission flux at different crop growth stages

**Figure 4:**
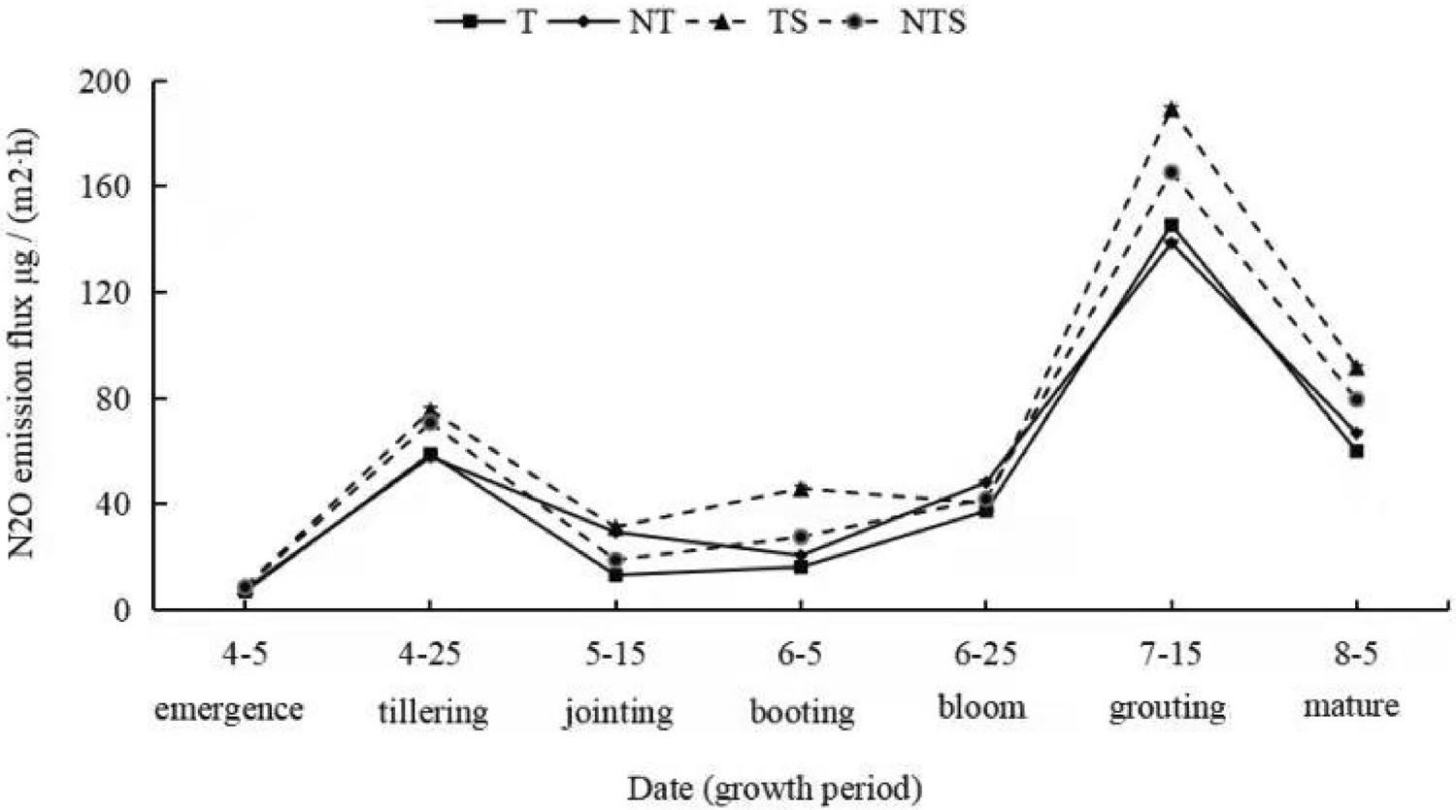
Soil N_2_O emission fluxes under different tillage measures.

Figure 4 shows the seasonal dynamics of N_2_O emission from spring wheat soil under different tillage measures. It can be seen from the figure that the variation of soil N_2_O emission flux of each treatment in the whole growth period of spring wheat was basically the same. After sowing, the emissions were low and rose gently thereafter. The first emission peak appeared around April 25, which may be related to the base fertilizer application and then fell steadily and stabilized until around July 15, the second emission peak. The largest peak was TS treatment (189.35 μg m^−2^ h^−1^) while the smallest peak was NT treatment (138.51 μg m^−2^ h^−1^). Subsequently, the emission flux of N_2_O decreased rapidly until harvest, which may be related to the gradual decrease of soil fertility and the imbalance of water and heat conditions.

### Effects of conservation tillage on soil N_2_O cumulative emission and yield

**Figure 5:**
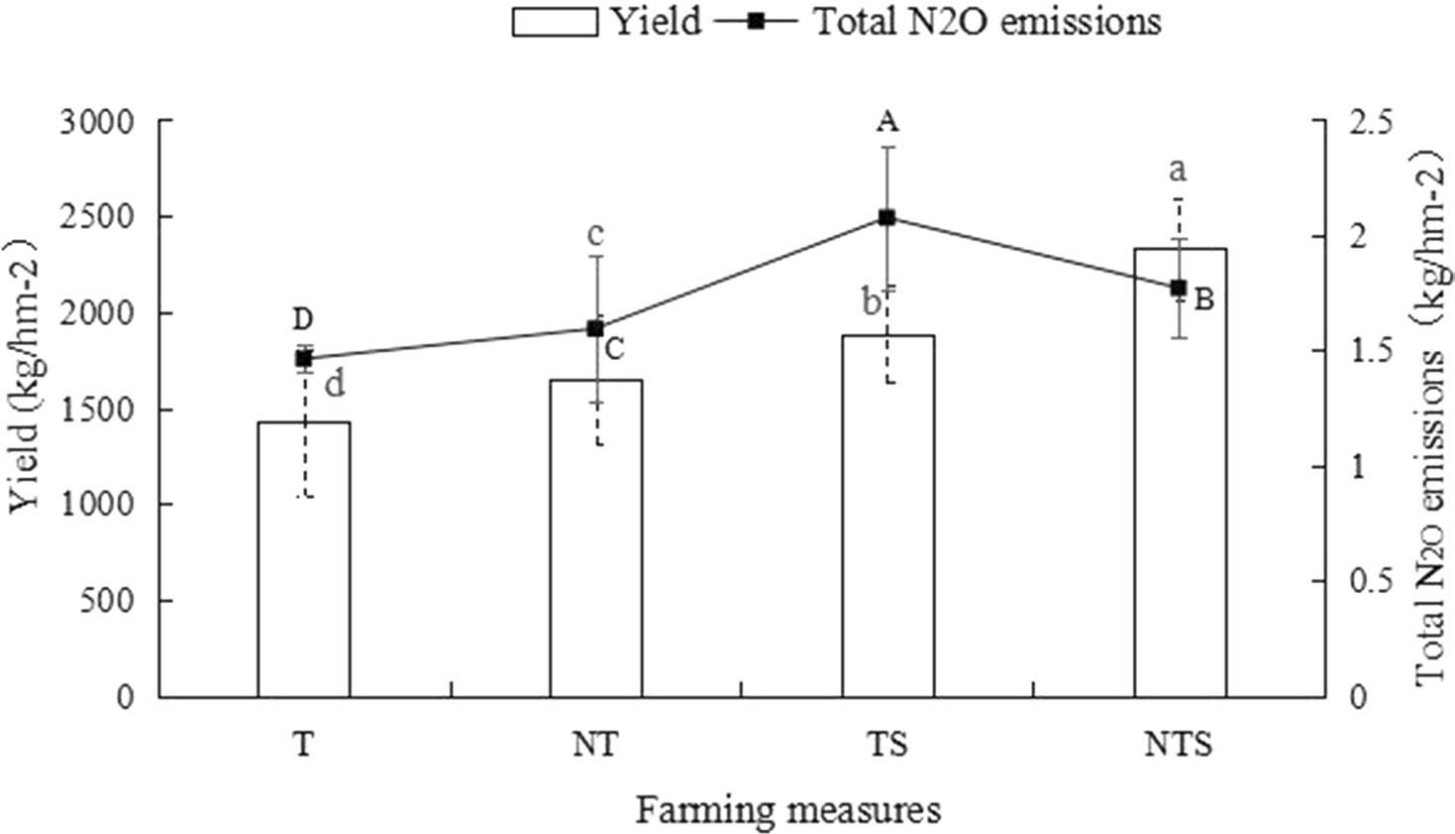
Spring wheat yield and total N_2_O emissions under different tillage measures. Different capital letters indicate that there are significant differences in total N_2_O emissions under different treatments; Different lowercase letters indicate that there are significant differences in yield under different treatments.

By analyzing the yield of spring wheat and the cumulative emission of soil N_2_O under each treatment (Fig. 5), it can be seen that the total N_2_O emission under the TS treatment is the largest, and the total N_2_O emission under the T treatment is the smallest. Among them, the total N_2_O emission under TS treatment was 41.9%, 17.22% and 30.24% higher than T, NT and NTS treatments respectively, and the difference between treatments was significant (*P* < 0.05). From the perspective of spring wheat yield, compared with T treatment, NT, TS and NTS treatments increased yield by 15.40%, 31.97% and 63.21%, indicating that conservation tillage can effectively increase crop yields, especially straw mulching.

### Correlation analysis between soil physical and chemical properties and N_2_O emission

**Figure 6:**
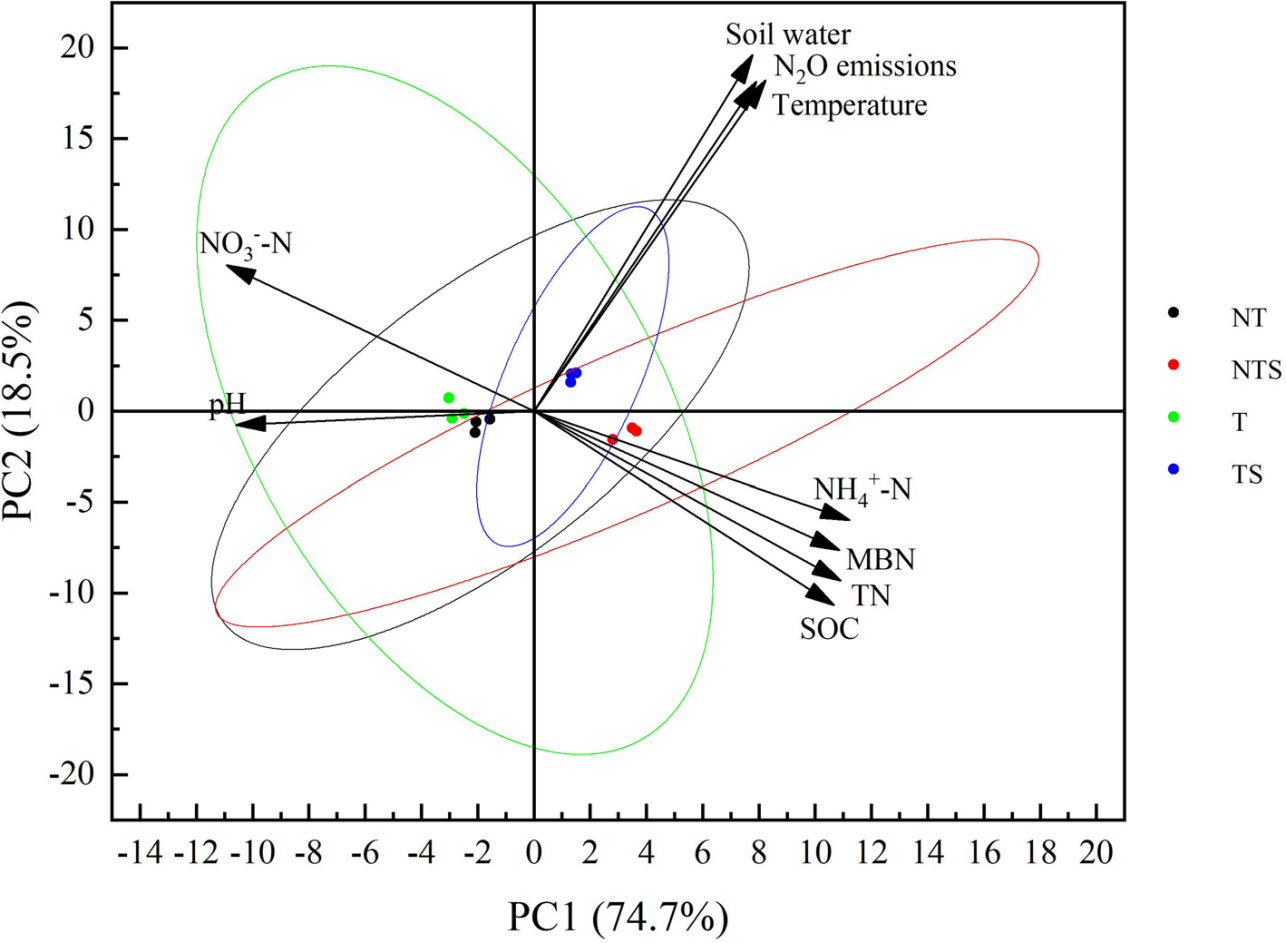
Principal component analysis of N_2_O emission and physical and chemical properties of soil.

**Figure 7:**
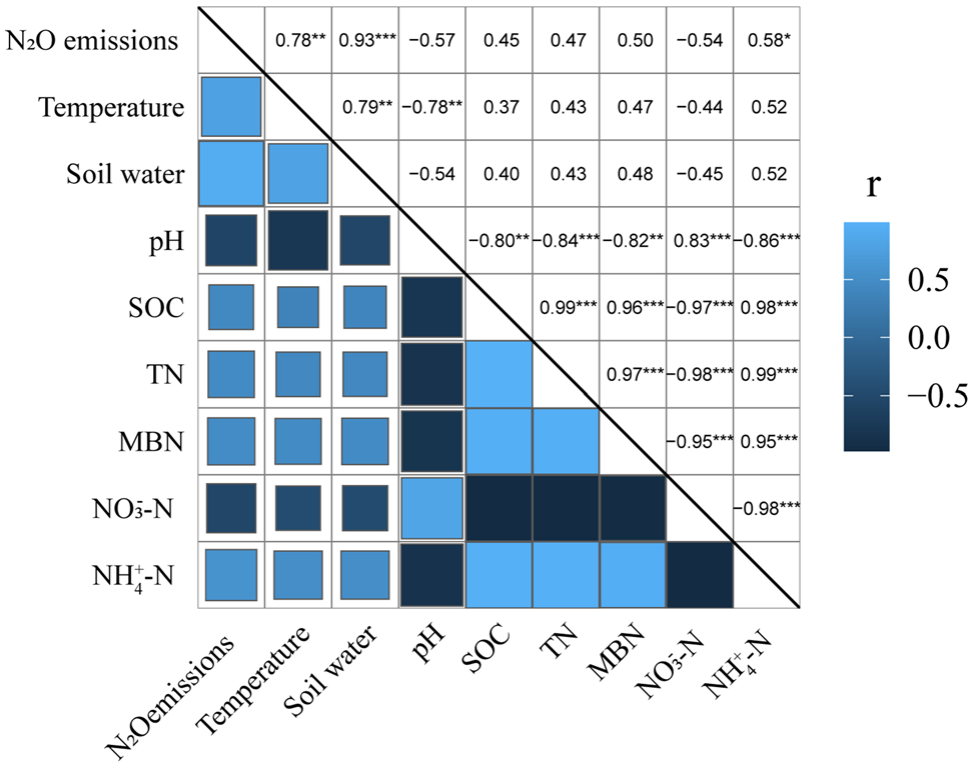
Correlation between N_2_O emissions and soil physical and chemical properties. *T* temperature. *Indicates significant at 0.05 level, **indicates significant at 0.01 level, ***indicates significant at 0.001 level.

The correlation between soil N_2_O emission and soil physical and chemical properties under different tillage measures is shown in Figs. 6 and 7. As can be seen from Fig. 6, the interpretation of the first ranking axis of principal component analysis was 74.7%, and the interpretation of the second ranking axis was 18.5%, which is mainly determined by the first ranking axis. At the same time, Fig. 7 also showed there was a very significant positive correlation between soil N_2_O emission and soil temperature and soil water content, and the correlation coefficients were 0.780 and 0.930 (Fig. 7). There was a significant positive correlation with NH_4_^+^-N, and the correlation coefficient was 0.582 (Fig. 7). There were also positive correlations with SOC, TN and MBN, nonetheless they were not significant (Fig. 7). There were also negative correlations with soil pH and NO_3_^–^N, with correlation coefficients of − 0.573 and − 0.535, but they were also not significant (Fig. 7).

## Discussion

### Effects of different tillage measures on soil physical properties

Previous studies have shown that conservation tillage has increased soil bulk density and pH compared with traditional tillage in South Asia rice and wheat planting system^30^. Another study showed that NT helped to increase soil bulk density in soybean fields in central India, and different tillage management could significantly affect soil physical properties^31^. However, our study found that in the whole growth period of spring wheat, conservation tillage increased soil bulk density and water content compared with traditional tillage. This is because traditional tillage increases the disturbance to the surface soil, destroys the structure of the soil plough layer, resulting in the loosening of the soil plough layer^32^, while conservation tillage reduces the disturbance to the soil plough layer, makes the soil compact, and thus increases the soil bulk density. In addition, due to the straw remaining in the field and less soil disturbance under no tillage, the aggregation of soil particles is enhanced, which effectively reduces evaporation and increases soil water storage^33^. On the other hand, straw mulching can reduce wind and water erosion, provide shade and improve soil moisture^34^. Therefore, the implementation of conservation tillage increases the soil bulk density and water content. In terms of temperature, in the early growth stage of spring wheat, the soil surface temperature of conservation tillage is higher than that of traditional tillage, because the conservation tillage based on no tillage and straw mulching can play a good role in heat preservation and moisture conservation. In the late growth stage of spring wheat, the soil surface temperature of conservation tillage is lower than that of traditional tillage. This is because under conservation tillage, spring wheat plants grow vigorously and can block most of the sunlight, thus reducing the surface temperature.

### Effects of conservation tillage on soil chemical properties

Studies have shown that in the dryland areas of the Loess Plateau, conservation tillage can increase crop productivity, increase soil nitrogen storage, and reduce soil nitrogen loss^35^. Studies have also shown that, compared with conventional tillage, conservation tillage significantly reduced the nitrogen loss of sloping farmland (especially the nitrogen loss due to surface runoff). In soils with poor nutrients, no-tillage is more effective than re-cultivation^36^. Our study found that during the growth period of spring wheat, conservation tillage increased soil organic matter and decreased soil pH value compared with traditional tillage. This is because traditional tillage increases the disturbance of surface soil, destroys the structure of soil plough layer, leads to the decline of soil fertility, the increase of soil porosity, the loss of nutrients, and finally the reduction of soil organic matter. On the other hand, no tillage and straw mulching can reduce wind and water erosion, provide shade and improve soil moisture, so as to reduce soil pH. In the middle and late growth stage, a large number of straws may be decomposed under the action of microbial decomposition, so as to increase the content of soil organic carbon^34^. Therefore, the implementation of conservation tillage increases soil organic matter and reduces soil pH.

Our study also found that the contents of soil nitrogen components under different tillage measures were also different. From the whole growth period, the contents of soil TN, MBN and NH_4_^+^-N under the three kinds of conservation tillage were higher than those under traditional tillage, which was the largest under NTS treatment. Also, the content of NO_3_^–^N under conservation tillage were lower than that under traditional tillage. This is because no-tillage reduced the direct impact of rain on the soil, reduced ground runoff and soil erosion, reduce ground evaporation, and well protected the original structure of the soil^37^. Secondly, compared with traditional tillage, no tillage reduced the compaction of soil and the damage to soil structure, makes the compactness of plough layer more appropriate, slows down the mineralization rate of soil organic matter, and is conducive to the accumulation of nutrients and the growth of crop roots; On the other hand, straw mulching can play a good role in water storage and moisture conservation. With the decay and decomposition of organic mulching materials, it can increase the content of soil carbon and nitrogen and improve the physical and chemical properties of soil^38^. In this study, the organic matter content in no tillage straw mulching treatment is significantly higher than that in traditional tillage, which also proves this conclusion (Table 5). The high residual NO_3_^–^N content in the soil surface under traditional tillage is due to the great destruction of the soil topsoil under traditional tillage, the loss of soil nutrients, the reduction of water content and the enhancement of soil aeration. NH_4_^+^-N is easier to be transformed into NO_3_^–^N, resulting in the increase of soil NO_3_^–^N content^39^. At the same time, compared with conservation tillage, spring wheat under traditional tillage has weak growth and poor root development, The ability to absorb nutrients into the soil is not strong, resulting in the residue of NO_3_^–^N^40^. This is similar to the results of Pisani et al. In the study of plain soil in the United States^41^.

### Effects of different tillage measures on N_2_O emission

Cao et al.^42^ found out that soil N_2_O emission basically occurs in the surface area of the soil, and the increase in soil water content caused by rainfall can directly affect soil N_2_O emission. Liu et al.^43^ in their study of greenhouse gas emissions from dryland wheat soil under no-tillage showed that N_2_O emissions have an exponential function relationship with surface temperature, 5 cm belowground temperature and air temperature. This study showed that dryland spring wheat fields are emission sources of N_2_O, and the N_2_O emission trends of each treatment were similar. There are two emission peaks during the growth period of spring wheat, which are around April 25 and July 15, respectively. These emission peaks may be due to the application of basal fertilizer during planting. By April 25, the temperature gradually increased compared to before planting. At this period soil freeze–thaw period was over, and so the effect of the basal fertilizer was then exerted, which increased soil microbial activity, causing the small emission peak. Subsequently, as the plants grew at seedling stage by utilizing the available nutrients, soil fertility and soil moisture reduced, leading to rapid decrease in N_2_O emission which remained at a low level. Afterwards, the second emission peak appeared on July 15 because on the one hand, due to the significant increase in rainfall (Table 2), the water content in the soil increased and the pH value of the soil decreased. At this time, soil temperature also increased, resulting in denitrification process^44^. Furthermore, the spring wheat was in its later stage of growth, where demand for nitrogen in the soil becomes less, and the surface litter returns part of the nitrogen to the soil through the decomposition. Therefore, the overall nitrogen content in the soil increased, further enhancing denitrification^42^, and subsequently the second N_2_0 emission peak.

### Effects of conservation tillage on soil N_2_O cumulative emission and yield

Studies have shown that soil N_2_O emission significantly increased after surface mulching^45,46^, but other studies found that plastic film (polyethylene) mulching and no-tillage combined with straw mulching both significantly reduced soil N_2_O flux^47^. Chen et al. Found that conservation tillage can reduce N2O emissions from vegetable fields when studying the vegetable ecosystem in the American plain^48^. But this study shows that compared with traditional tillage, conservation tillage actually increased N_2_O emissions, especially under TS treatment. N_2_O emission followed the trend as follows: TS > NTS > NT > T. This is because the decomposition of straw into soil under straw mulching increased the input of soil organic matter, which accumulates in the soil and increases the carbon in the soil. This increase in soil mineral nutrients changes soil properties, and enhances the activities of nitrifying and denitrifying bacteria. Additionally, straw mulching induces heat and conserves moisture, providing suitable temperature and moisture conditions for nitrification and denitrification^28^. Most studies have shown that straw mulching can alter soil N_2_O emission rate by affecting soil moisture and soil nutrient status^49,50^. The lowest N_2_O emission under traditional tillage is because traditional tillage greatly destroys the soil plough layer, resulting in increased soil porosity, weakened soil fertility, nutrient loss, inhibition of microbial activity, which are not conducive for the accumulation of soil nitrification and denitrification substrates, hence reduction in N_2_O emission.

### Relationship between soil physicochemical properties and N_2_O emission

In drylands, soil temperature is a key factor affecting seasonal variations of N_2_O emissions. Bremner et al.^51^ found that the change of N_2_O emission rate is almost synchronized with the surface soil temperature. Maag et al.^52^ found that increasing soil temperature can reduce the contribution of nitrification to N_2_O and increase the amount of N_2_O produced during denitrification. Correlation analysis of this experiment showed that soil N_2_O emissions have a very significant positive correlation with soil temperature. This is because the increase in microbial respiration after warming causes oxygen deficiency in the soil profile which creates anaerobic condition for denitrifying microorganisms’ activity^53^, and at the same time warming also increases soil denitrification activity^54^, which leads to an increase in N_2_O emissions. Water affects the metabolic activity of soil microbial cells and the transportation of nutrients, and has a decisive influence on the process of nitrification and denitrification. Hu et al.^55^ believe that the formation of soil "hot spots" is mainly because the increase in water content increases the enrichment of soil available carbon and nitrogen, which in turn affects the distribution of oxygen concentration in the soil. In a soil environment where oxygen is limited and available carbon is abundant, denitrification is the dominant channel for N_2_O emissions in the "hot spot" area. The results of this experiment shows that there is a very significant positive correlation between soil N_2_O emissions and soil moisture, which is consistent with the results of previous studies. This is because the increase in soil moisture will limit the oxygen concentration of the soil, leading to the formation of an anaerobic soil environment, significantly reducing nitrification and enhancing denitrification, which in turn leads to denitrification as the dominant way to produce N_2_O emissions^56^. This experimental study also found that soil N_2_O emissions are positively correlated with soil SOC, TN, MBN and NH_4_^+^-N content. This is because the increase in soil SOC, TN, MBN and NH_4_^+^-N content will provide sufficient nitrogen sources. It will significantly increase the respiration of heterotrophic microorganisms in the soil. At the same time, the available carbon and available nitrogen in the soil provide electron donors for denitrifying microorganisms, which promotes the occurrence of denitrification, thereby increasing N_2_O emissions. Additionally, soil N_2_O emissions are negatively correlated with soil NO_3_^–^N and soil pH. This is because a large amount of precipitation enters the soil during the rainy season, which increases soil moisture and converts soil NO_3_^–^N to NH_4_^+^-N, and the soil becomes a low-oxygen environment, which provides the most suitable conditions for denitrification and greatly increases N_2_O emissions^57^. Studies have shown that pH is positively correlated with N_2_O produced by the denitrification process of nitrifying bacteria, and negatively correlated with N_2_O produced by denitrification. Cao et al.^42^ found that increasing soil pH would increase soil denitrification rate and promote the reduction of N_2_O to N_2_, this is consistent with the results found in this study, that is in an alkaline environment, the main product of denitrification is N_2_, and the amount of N_2_O produced is small. The main reason is that low pH will interfere with N_2_O reductase assembly (no gene expression) during denitrification, reduce enzyme activity, and increase N_2_O emissions from denitrification^58^.

## Conclusions

This study provided evidence for the response of soil N_2_O emissions from spring wheat fields in the Loess Plateau to different farming methods by studying the physical and chemical properties of farmland soil, nitrogen content and N_2_O emission flux. The results showed that compared with traditional tillage, conservation tillage with no tillage and straw mulching significantly increased surface soil bulk density, organic carbon and soil water content, and reduced soil pH value; For soil nitrogen components, the three conservation tillage increased the contents of soil total nitrogen, microbial biomass nitrogen and ammonium nitrogen, and decreased the residue of soil nitrate nitrogen. Our data also shows that conservation tillage increases soil N_2_O emissions compared to traditional tillage, the correlation shows that soil physical and chemical properties and nitrogen content have a certain impact on N_2_O emissions. Among them, soil temperature and water content are the most significant factors affecting N_2_O emissions. We can further reduce N_2_O emissions by regulating soil temperature and water content. Additionally, NTS treatment greatly increased crop yield compared with traditional tillage, but did not significantly increase soil N_2_O emissions. Consequently, we have comprehensive economic and environmental benefits and recommend no-tillage mulch (NTS) as the most suitable tillage measure in the study area. In general, this study provides relevant information on farmland soil nitrogen fixation and emission reduction, which helps us better explore the impact mechanism of different tillage measures on farmland soil nitrogen components and N_2_O emissions, which may further provide positive feedback for coping with climate change. Whereas, considering the joint effects of climate factors, soil microorganisms and enzyme activities on farmland soil and greenhouse gas emissions, we still need long-term, systematic and comprehensive research to better understand the dynamic change mechanism of farmland soil physical and chemical properties and greenhouse gas emissions under different tillage measures.

## References

[1] Fu CH (2015). Relationships among fisheries exploitation, environmental conditions, and ecological indicators across a series of marine ecosystems. J. Mar. Syst..

[2] Too CC, Ong KS, Yule CM, Keller A (2020). Putative roles of bacteria in the carbon and nitrogen cycles in a tropical peat swamp fores. Basic Appl. Ecol..

[3] Hou RJ (2020). Effects of biochar and straw on greenhouse gas emission and its response mechanism in seasonally frozen farmland ecosystems. Catena.

[4] Wang X, Lu P, Yang PL, Ren SM (2021). Effects of fertilizer and biochar applications on the relationship among soil moisture, temperature, and N_2_O emissions in farmland. PeerJ.

[5] Tang ZM, Liu XR, Zhang QW, Li GC (2021). Effects of biochar and straw on soil N_2_O emission from a wheat maize rotation system. Huan Jing Ke Xue.

[6] Kong Q, Wang ZB, Niu PF, Miao MS (2016). Greenhouse gas emission and microbial community dynamics during simultaneous nitrification and denitrification process. Biores. Technol..

[7] Han ZM (2021). Spatial-temporal dynamics of agricultural drought in the Loess Plateau under a changing environment: Characteristics and potential influencing factors. Agric. Water Manag..

[8] Clemens S (2017). Nitrification inhibitors can increase post-harvest nitrous oxide emissions in an intensive vegetable production system. Sci. Rep..

[9] Zhang DJ (2021). Effects of tillage and fertility on soil nitrogen balance and greenhouse gas emissions of wheat-maize rotation system in Central Henan Province, China. J. Appl. Ecol..

[10] Liu XC (2014). Response of soil N_2_O emissions to precipitation pulses under different nitrogen availabilities in a semiarid temperate steppe of Inner Mongolia, China. J. Arid Land.

[11] Hu QY (2019). Combined effects of straw returning and chemical n fertilization on greenhouse gas emissions and yield from paddy fields in northwest Hubei Province, China. J. Soil Sci. Plant Nutr..

[12] Sun ZC (2019). Effects of straw returning and feeding on greenhouse gas emissions from integrated rice-crayfish farming in Jianghan Plain, China. Environ. Sci. Pollut. Res..

[13] Mei K (2018). Stimulation of N_2_O emission by conservation tillage management in agricultural lands: A meta-analysis. Soil Tillage Res..

[14] Wang HY, Wu JQ, Li G, Yan LJ (2020). Changes in soil carbon fractions and enzyme activities under different vegetation types of the northern Loess Plateau. Ecol. Evol..

[15] Sadiq M, Li G, Rahim N, Tahir MM (2021). Sustainable conservation tillage technique for improving soil health by enhancing soil physicochemical quality indicators under wheat mono-cropping system conditions. Sustainability.

[16] Nie ZG (2021). Evaluating the effects of different sowing dates and tillage methods on dry-land wheat grain dry matter accumulation based on the APSIM model. J. Appl. Ecol..

[17] Alhassan AM, Yang CJ, Ma WW, Li G (2021). Influence of conservation tillage on Greenhouse gas fluxes and crop productivity in spring-wheat agroecosystems on the Loess Plateau of China. PeerJ.

[18] Mou LM (2015). Breeding report of a new dryland spring wheat variety Dingxi 42. Gansu Agric. Sci. Technol..

[19] Ma WW, Li G, Wu JH, Xu GR, Wu JQ (2020). Respiration and CH_4_ fluxes in Tibetan peatlands are influenced by vegetation degradation. CATENA.

[20] Wu JQ (2020). Vegetation degradation impacts soil nutrients and enzyme activities in wet meadow on the Qinghai-Tibet Plateau. Sci. Rep..

[21] Défossez P (2021). Impact of soil water content on the overturning resistance of young Pinus Pinaster in sandy soil. For. Ecol. Manag..

[22] Mao J, Nierop KG, Rietkerk M, Damsté JSS, Te Dekker SC (2016). infuence of vegetation on soil water repellency-markers and soil hydrophobicity. Sci. Total Environ..

[23] Lu Y, Si B, Li H, Biswas A (2019). Elucidating controls of the variability of deep soil bulk density. Geoderma.

[24] Huang TT, Yang N, Lu C, Qin XL, Siddique K (2021). Soil organic carbon, total nitrogen, available nutrients, and yield under different straw returning methods. Soil Tillage Res..

[25] Yang JM, Zhang ZQ, Cao GJ (2014). Soil nitrate and nitrite content determined by Skalar SAN^++^. Soil Fertil. Sci. China.

[26] Chen N (2021). Effect of biodegradable film mulching on crop yield, soil microbial and enzymatic activities, and optimal levels of irrigation and nitrogen fertilizer for the Zea mays crops in arid region. Sci. Total Environ..

[27] Akhtar K (2020). Straw mulching with inorganic nitrogen fertilizer reduces soil CO_2_ and N_2_O emissions and improves wheat yield. Sci. Tot. Environ..

[28] Ma E (2009). Effects of rice straw returning methods on N_2_O emission during wheat-growing season. Nutr. Cycl. Agroecosyst..

[29] Yeboah S (2016). Greenhouse gas emissions in a spring wheat–field pea sequence under different tillage practices in semi-arid Northwest China. Nutr. Cycl. Agroecosyst..

[30] Zahid A, Ali S, Ahmed M, Iqbal N (2020). Improvement of soil health through residue management and conservation tillage in rice-wheat cropping system of Punjab, Pakistan. Agronomy.

[31] Dharmendra S (2020). Effect of reversal of conservation tillage on soil nutrient availability and crop nutrient uptake in soybean in the vertisols of central India. Sustainability..

[32] Orzech K, Wanic M, Załuski D (2021). The effects of soil compaction and different tillage systems on the bulk density and moisture content of soil and the yields of winter oilseed rape and cereals. Agriculture.

[33] Fan BQ, Liu QL (2005). Effect of conservation tillage and straw application on the soil microorganism and P-dissolving characteristics. Chin. J. Eco-Agric..

[34] Liu X (2019). Dynamic contribution of microbial residues to soil organic matter accumulation influenced by maize straw mulching. Geoderma.

[35] Wang WY (2020). Conservation tillage enhances crop productivity and decreases soil nitrogen losses in a rainfed agroecosystem of the Loess Plateau, China. J. Clean. Prod..

[36] Zhang Y, Xie DT, Ni JP, Zeng XB (2020). Conservation tillage practices reduce nitrogen losses in the sloping upland of the Three Gorges Reservoir area: No-till is better than mulch-till. Agric. Ecosyst. Environ..

[37] Andrea F (2020). May conservation tillage enhance soil C and N accumulation without decreasing yield in intensive irrigated croplands? Results from an eight-year maize monoculture. Agric. Ecosyst. Environ..

[38] Wu J (2019). Effects of different tillage and straw retention practices on soil aggregates and carbon and nitrogen sequestration in soils of the northwestern China. J. Arid. Land.

[39] Niu, Y. N., Shen, Y. Y., Nan, Z. B., Yang, J. & Yang, Z. W. College of Pastoral Agriculture Science & Technology, Lanzhou University, China. Influence of different cultivation managements on organic carbon and nitrate nitrogen of top soil in the Loess Plateau, northwestern China. *Proceedings of the XXI International Grassland Congress and the VIII International Rangeland Congress (volume II)* (2008).

[40] Wang Q, Li FR, Zhang EH, Li G, Vance M (2012). The effects of irrigation and nitrogen application rates on yield of spring wheat (longfu-920), and water use efficiency and nitrate nitrogen accumulation in soil. Aust. J. Crop Sci..

[41] Pisani O (2017). Soil nitrogen dynamics and leaching under conservation tillage in the Atlantic Coastal Plain, Georgia, United States. J. Soil Water Conserv..

[42] Cao WC (2019). Key production processes and influencing factors of nitrous oxide emissions from agricultural soils. J. Nutr. Fertil..

[43] Liu B, Huang GB, Gao YQ, Li QP, Huang T (2010). Effects of no-tillage on daily dynamics of CO_2_ and N_2_O emission from spring wheat field during mature stage. J. Gansu Agric. Univ..

[44] Akhtar K (2020). Straw mulching with inorganic nitrogen fertilizer reduces soil CO_2_ and N_2_O emissions and improves wheat yield. Sci. Total Environ..

[45] Sina B, Youngsun K, Janine K, Gerhard G (2013). Plastic mulching in agriculture: Friend or foe of N_2_O emissions. Agric. Ecosyst. Environ..

[46] Seiichi N, Michio K, Masako T, Seiichiro Y, Naoto K (2012). Nitrous oxide evolved from soil covered with plastic mulch film in horticultural field. Biol. Fertil. Soils.

[47] Wang J, Cai LQ, Zhang RZ, Wang YL, Dong WJ (2011). Effects of Tillage Measures on soil greenhouse gas (CO_2_, CH_4_, N_2_O) flux in temperate semi-arid area. Chin. J. Eco-Agric..

[48] Chen GH (2018). Can conservation tillage reduce N_2_O emissions on cropland transitioning to organic vegetable production?. Sci. Total Environ..

[49] Narendra KL, Rattan L (2013). Soil aggregation and greenhouse gas flux after 15 years of wheat straw and fertilizer management in a no-till system. Soil Tillage Res..

[50] Liang W, Shi Y, Zhang H, Yue J, Huang GH (2007). Greenhouse gas emissions from Northeast china rice fields in fallow season. Pedosphere.

[51] Bremner JM, Robbins SG, Blackmer AM (1980). Seasonal variability in emission of nitrous oxide from soil. Geophys. Res. Lett..

[52] Maag M, Vinther FP (1996). Nitrous oxide emission by nitrification and denitrification in the different soil types and at different soil moisture contents and temperature. Appl. Soil. Ecol..

[53] Castaldi S (2000). Responses of nitrous oxide, dinitrogen and carbon dioxide production and oxygen consumption to temperature in forest and agricultural light-textured soils determined by model experiment. Biol. Fertil. Soils.

[54] Braker G, Schwarz J, Conrad R (2010). Influence of temperature on the composition and activity of denitrifying soil communities. FEMS Microbiol. Ecol..

[55] Hu HW, Chen D, He JZ (2015). Microbial regulation of terrestrial nitrous oxide formation: Understanding the biological pathways for prediction of emission rates. Narnia.

[56] Pokharel P, Chang SX (2021). Biochar decreases the efficacy of the nitrification inhibitor nitrapyrin in mitigating nitrous oxide emissions at different soil moisture levels. J. Environ. Manage..

[57] Shu XX (2021). Response of soil N_2_O emission and nitrogen utilization to organic matter in the wheat and maize rotation system. Sci. Rep..

[58] Bergaust L, Mao YJ, Bakken LR, Frostegård A (2010). Denitrification response patterns during the transition to anoxic respiration and posttranscriptional effects of suboptimal pH on nitrous [corrected] oxide reductase in *Paracoccus denitrificans*. Appl. Environ. Microbiol..

